# FOXO3-induced lncRNA LOC554202 contributes to hepatocellular carcinoma progression via the miR-485-5p/BSG axis

**DOI:** 10.1038/s41417-021-00312-w

**Published:** 2021-03-02

**Authors:** Lin Yang, Wan-li Deng, Bao-guo Zhao, Yao Xu, Xiao-wen Wang, Yu Fang, Hai-juan Xiao

**Affiliations:** 1 Department of Hepatobiliary Surgery, The Xianyang Central Hospital, Xianyang, Shaanxi China; 2 Department of Oncology, The Putuo Hospital, Shanghai University of Traditional Chinese Medicine, Putuo, Shanghai China; 3 Department of Oncology, The Affiliated Hospital of the Shaanxi University of Traditional Chinese Medicine, Xianyang, Shaanxi China

**Keywords:** Oncogenes, Biomarkers

## Abstract

Long non-coding RNAs (LncRNAs) have played very important roles in the malignancy behaviors of hepatocellular carcinoma (HCC). LncRNA LOC554202 (LOC554202) was a newly identified tumor-related lncRNA. However, its expression and function in HCC remained unknown. In this study, we firstly reported that LOC554202 expression was distinctly upregulated in HCC specimens and cell lines. Clinical assays indicated that increased LOC554202 expression had a diagnostic value for HCC patients and was positively associated with advanced stages and poor clinical prognosis. Additionally, forkhead box O3(FOXO3) could bind directly to the LOC554202 promoter region and activate its transcription. Functionally, we observed that knockdown of LOC554202 suppressed the proliferation, migration, invasion, and epithelial–mesenchymal transition (EMT) progress of HCC cells, and promoted apoptosis. Mechanistically, LOC554202 competitively bound to miR-485-5p and prevented the suppressive effects of miR-485-5p on its target gene basigin (BSG), which finally led to HCC metastasis, EMT, and docetaxel chemoresistance. Our data demonstrated that FOXO3-induced LOC554202 contributed to HCC progression by upregulating BSG via competitively binding to miR-485-5p, which suggested that the regulation of the FOXO3/LOC554202/miR-485-5p/BSG axis may have beneficial effects in the treatment of HCC.

## Introduction

Hepatocellular carcinoma (HCC) acts as a common malignant disease and the major reason for death resulted from cancer worldwide with its incidence rapidly increasing in recent years [1]. It was reported that the number of new HCC cases and HCC death in the People’s Republic of China (China) occupied 50% of the total in the world [2]. A lot of risk factors can lead to HCC, particularly the hepatitis B and C viruses as well as liver cirrhosis [3]. Despite progress in treatment protocols, including precious hepatectomy and liver transplantation, a 5-year overall survival (OS) for HCC is <5% because of its high recurrence and metastasis rate [4, 5]. Under such background, researchers are suggested to figure out the underlying molecular mechanisms of HCC and seek for new biomarkers to screen and diagnose HCC in an early stage.

In recent studies, different genome-wide methods including the ENCODE project have been combined, finding the transcription of most mammalian genomes, with only about 1.5% representing protein-coding genes [6]. Long non-coding RNAs (lncRNAs) belong to a class of transcribed RNA with a length of over 200 nucleotides, which distinguishes them from small non-coding RNAs, such as miRNA, tRNA, and rRNA [7]. Due to the lack of protein-coding sequences, ncRNAs are the transcripts without coding potential. However, more and more evidence has revealed that lncRNAs participate in the regulatory processes of chromatin modification, transcriptional interference, transcriptional activation, selective splicing, and regulation of tumor-related gene activation, so as to regulate gene expression at epigenetic, transcriptional, or posttranscriptional levels [8, 9]. Moreover, growing studies have demonstrated that lncRNAs, due to the special profiles in different human cancers, stand for the progress of diseases and can be used to predict the diagnosis and prognosis [10, 11]. However, the functions of a large number of lncRNAs were still unclear in HCC.

Although more and more dysregulated lncRNAs have been identified in various tumors, the potential mechanisms remained unclear. In recent years, growing evidence suggested that transcription factors (TFs) could activate the transcription of lncRNAs [12, 13]. Forkhead box O3 (FOXO3) belongs to the O subclass of the forkhead family of transcription factors and has been reported to exhibit multiple effects in tumors [14, 15]. In HCC, FOXO3 has been reported to be distinctly upregulated and promote tumor progression [16, 17]. However, whether FOXO3 could regulate the expression of lncRNAs was rarely reported.

LncRNA LOC554202 (LOC554202), located on 9p21.3, was a recently identified lncRNA related to tumors with a length of 2166 nt. In recent years, the distinct dysregulation of LOC554202 and its tumor-related effects have been reported in several tumors, such as breast cancer, gastric carcinoma, and colorectal carcinoma [18–20]. In gastric carcinoma, LOC554202 was shown to promote the proliferation and migration of gastric carcinoma cells via regulating p21 and E-cadherin [19]. In lung cancer and laryngeal squamous cell carcinoma, LOC554202 was also shown to serve as a tumor promoter [21, 22]. Nevertheless, no studies have reported its expression and biological function in HCC.

## Materials and methods

### Patients and specimens

We collected 137 primary HCC specimens and corresponding non-tumor specimens in total from the Xianyang Central Hospital for RT-qPCR analysis between July 2014 and March 2017. Patients’ ages ranged from 28 to 69 years, and the average age was (49.32 ± 12.51) years. All patients recruited in this study did not receive chemotherapy and/or preoperative radiotherapy. All the patients were followed up every three months by telephone until death or the end of follow-up. Study approval was obtained from independent Ethics Committees at the Xianyang Central Hospital. All participants provided written consent.

### Cell culture and cell transfections

We obtained human 293T cells, human HCC cell lines (including HepG2, Hep3B, Huh7, SMMC-7721, and LM3), and human hepatocyte cell line (LO2) cells from the Type Culture Collection of the Chinese Academy of Sciences (Shanghai, China). All cells were cultured in Dulbecco’s modified Eagle’s medium (DMEM, Gibco, Guangzhou, Guangdong, China) that contained fetal bovine serum (FBS, 10%, Gibco, Guangzhou, Guangdong, China). 100 units/mL penicillin (Shenhu Technology, Pudong, Shanghai, China) and 100 μg/mL streptomycin (Sigma, Nanjing, Jiangsu, China) are supplemented.

LOC554202 small interfering RNA (si-LOC554202-#1 and si-LOC554202-#2, GenePharma, Huixu, Shanghai, China) along with corresponding control RNA (siRNA NC), or recombinant plasmid overexpressing FOXO3 (pcDNA-FOXO3) along with empty pcDNA3.1 vector (MBL, Technology, Hangzhou, Zhejiang, China), or miR-485-5p mimics (Santa, Kunming, Yunan, China) along with corresponding control RNA (mimics NC), or miR-485-5p inhibitor (anti-miR-485-5p) and inhibitor negative control (anti-miR-NC) were transfected into cells. The Lipofectamine 2000 transfection reagent (Invitrogen, Hangzhou, Zhejiang, China) helped to conduct the transfection following the protocol of the manufacturer.

### Quantitative real-time PCR (qRT-PCR)

The implementation steps of qRT-PCR analysis were based on the previous description [23]. Primers for RT-RCR are presented in Table 1.

**Table 1:** The primers used in this study for RT-PCR.

Names	Sequences (5′–3′)
LOC554202: F	TCTCTGGTGCTTCCCTCCTT
LOC554202: R	GATCTAAGCTTGAGCCCCCA
FOXO3: F	GCGTGCCCTACTTCAAGGATAAG
FOXO3: R	GACCCGCATGAATCGACTATG
miR-485-5p: F	CCAAGCTTCACCCATTCCTAACAGGAC
miR-485-5p: R	CGGGATCCGTAGGTCAGTTACATGCATC
BSG: F	CAGAGTGAAGGCTGTGAAGTCG
BSG: R	TGCGAGGAACTCACGAAGAA
GAPDH: F	CAAGGTCATCCATGACAACTTTG
GAPDH: R	GTCCACCACCCTGTTGCTGTAG
U6: F	CTCGCTTCGGCAGCACA
U6: R	AACGCTTCACGAATTTGCGT

### Cell proliferation assays

HCC cells (3 × 10^3^ cells) received 1 day of incubation by using culture medium (120 μL) in 96-multiwell plates at 37 °C in 5% CO_2_. The plasmid was used to transfect cells for 24, 48, 72, and 96 h. Specifically, each well was added with Cell Counting Kit-8 (CCK-8) assay solution (10 mL), followed by 2 h of incubation. The microplate reader read the optical density exhibited by each well at 450 nm to estimate cell proliferation.

During the colony-formation assay, we placed 500 Huh7 and LM3 cells in total in a fresh six-well plate as well as maintained them in RPMI-1640 medium (Pierce, Guangzhou, Guangdong, China) that contained 10% FBS, and then replaced the medium every 3 days. Twelve days later, we used methanol to fix cells and used 0.1% crystal violet (Sigma-Aldrich) to stain them. The resulting solution was then photographed.

### 5-Ethynyl-2′-deoxyuridine (EdU) assays

5-Ethynyl-2′-deoxyuridine assays assisted in determining the cellular proliferation by the use of a KeyFluor532 Click-iTEdU kit (KeyGEN, Hangzhou, Zhejiang, China) based on the instruction of the manufacturer. In brief, cells were first transfected by using corresponding vector cells, followed by 2 h of incubation by using EdU at 37 °C. Then, 4% polyformaldehyde that contained phosphate buffer saline (PBS) was used for fixing these cells. Finally, fluorescence microscopy assisted in visualizing cells. We repeated experiments no less than three times.

### Flow cytometry assay

Huh7 and LM3 cells were transiently transfected with the related factors. Flow cytometry (FACScan; BD Biosciences) was conducted to analyze cells by virtue of CellQuest software (BD Biosciences, Beijing, Haidian, China). Flowjo V10 software (Tree Star, Shenzhen, Guandong, China) assisted in analyzing all the apoptosis data regarding various cell lines.

### Subcellular fractionation location

The PARIS Kit (Life Technologies, Hangzhou, Zhejiang, China) helped to separate nuclear from cytosolic fractions based on the instruction of the manufacturer.

### Transwell invasion assays

The manufacturer guideline (BD Bioscience, Nanjing, Jiangsu, China) was prepared with the 48-well BD BioCoat Matrigel Invasion Chambers (Nanjing, Jiangsu, China). We added 3 × 10^5^ cells to the upper wells which were separated by a PET membrane with a pore size of 8 μm that had a thin layer of matrigel basement membrane matrix. The lower chamber was added with a certain amount of FBS medium (600 μL, 10%). After cells received 24 h of incubation, 20% Giemsa was used to fix and stain those that migrated or invaded to membrane’s lower side. Then 85% ethanol and crystal violet staining were used to fix and stain filters, respectively. An inverted microscope (Olympus, Tokyo, Japan) helped to count the five random fields in each chamber.

### Wound-healing assay

Cells were cultured in a 6-well plate for 12 h and then wounded by a sterilized pipet tip to make a straight scratch. After cells were washed with PBS gently, 0.5% FBS together with 1% penicillin/streptomycin was used to culture cells in RPMI-1640 medium. Pictures were taken by an Olympus digital camera at 0 h and 24 h after wounding.

### Biotinylated RNA-pulldown assay

The conduction of RNA-pulldown followed the previous description [24].

### Chromatin immunoprecipitation (ChIP) assays

The EZ-Magna ChIP kit (Millipore, Pudong, Shanghai, China) was adopted in the ChIP assays for exploring the FOXO3 binding to the endogenous LOC554202 promoter. In brief, GC cells were cross-linked in 1% formaldehyde solution for 10 min at room temperature, followed by the addition of 130 mM of glycine for 8 min. Sonication helped to generate DNA fragments in the range of 200–300 bp. Antibodies of anti-FOXO3 (#9342, Cell Signaling Technology, Haidian, Beijing, China) and IgG were employed for each immunoprecipitation. The qRT-PCR analysis was conducted on input DNAs after immunoprecipitation.

### Luciferase assay

JASPAR (http://jaspar.genereg.net/) was used to identify the FOXO3-binding motif in the promoter region of LOC554202. The different fragment sequences were synthesized and then inserted into a pGL3-basic vector (Promega, Pudong, Shanghai, China). The putative targets of LOC554202 were predicted by the starbase 2.0. The reporter vector pmiRGLO-LOC554202-wild-type (LOC554202-WT) or miRGLO- LOC554202-mutant (LOC554202-MuT) containing the predicted miR-485-5p binding sites were purchased from GenePharma (Pudong, Shanghai, China). The reporter vector pmiRGLO-BSG-wildtype1(BSG-WT1), miRGLO-BSG-mutant1(BSG-MuT1), pmiRGLO-BSG-wildtype2(BSG-WT2), or miRGLO-BSG-mutant2(BSG-MuT2) containing the predicted miR-485-5p binding sites were purchased from GenePharma (Pudong, Shanghai, China). A luciferase assay kit (Promega, Madison, WI, USA) assisted in conducting the Luciferase assays based on the protocol of the manufacturer, according to the previous description [25].

### Western blot

Frozen tissues were lysed in the lysis buffer, and the BCA protein assay kit (Beyotime, Pudong, Shanghai, China) assisted in measuring the concentration of protein. 12% SDS-PAGE gel was used to separate proteins which were then transferred to the PVDF membranes (polyvinylidene difluoride; Bio-Rad). After 2 h of blocking with 5% skim milk, the membrane received 2 h of incubation with primary antibodies and 1 h of incubation with the secondary antibody at room temperature. ImageJ software (National Institutes of Health, Bethesda, MD, USA) helped to analyze the western blot results. Primary antibodies of E-cadherin (ab241677, Epitomics Inc., Burlingame, CA, USA), Vimentin (ab45939, Epitomics Inc., Burlingame), N-cadherin (ab76011, Epitomics Inc., Burlingame), GAPDH (ab8245, Abcam Inc.), and BSG (ab188190, Epitomics Inc., Burlingame) were used in the immunoblotting assays.

### Statistical analysis

SPSS statistical software package (standard version 18.0, SPSS Inc., Chicago, IL, USA) assisted in conducting the statistical analysis. Investigators were blinded to the sample group allocation during the experiment and analysis. Comparisons between the two groups were carried out by conducting *t*-test or chi-square test. Data among multiple groups were analyzed by a one-way analysis of variance (ANOVA), followed by a Tukey’s post hoc test. Receiver-operating characteristic (ROC) curves were used to evaluate the performance of FOXO3, LOC554202, miR-485-5p, and BSG to discriminate HCC specimens from non-tumor tissues. The Kaplan–Meier method and the log-rank test were carried out for plotting the survival plots. Univariate and multivariate analyses were conducted regarding the prognostic significance of LOC554202 by virtue of the Cox proportional hazards regression model. All data are presented as the means ± SEM or SD, and *p*-value < 0.05 was considered with statistical significance.

## Results

### The distinct upregulation of LOC554202 and its diagnostic value in HCC patients

**Fig. 1:**
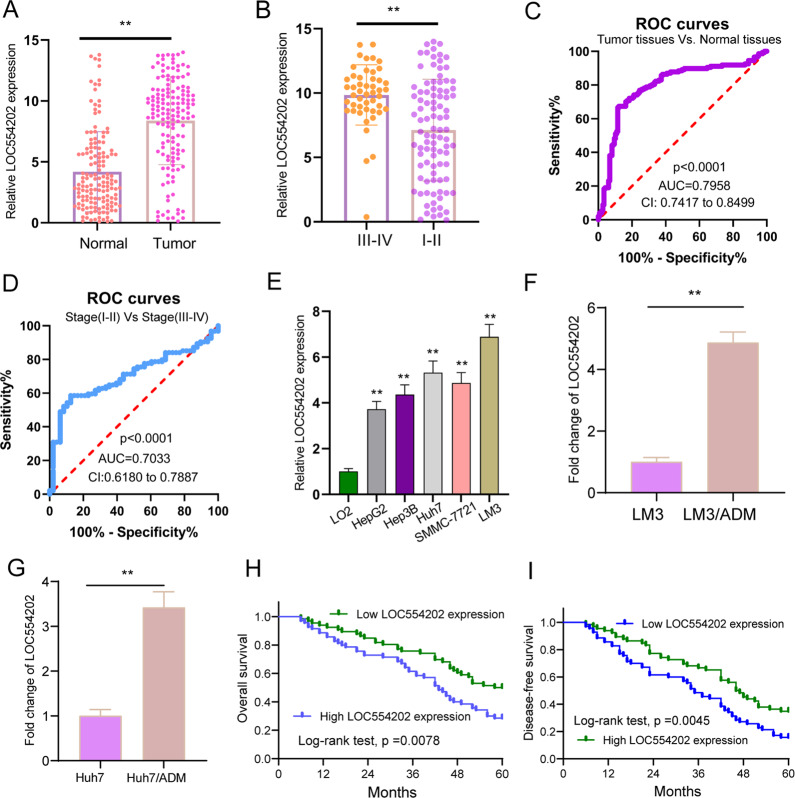
The distinct upregulation of LOC554202 in HCC patients and its clinical significance. **A** RT-PCR for the expression of LOC554202 in HCC specimens and matched non-tumor specimens. **B** The expression of LOC554202 in advanced grades using RT-PCR. **C** ROC curves of tissue LOC554202 expression for differentiating HCC tissue from normal tissue. **D** ROC curves of tissue LOC554202 expression for differentiating advanced HCC tissue from early HCC tissue. **E** Relative expression of LOC554202 in five HCC cell lines and human normal liver cell (LO2). **F**, **G** The levels of LOC554202 were determined in LM3/ADM and Huh7/ADM cells by RT-PCR. **H**, **I** Kaplan–Meier curves of the overall survival and disease-free survival of 137 HCC patients. *p < 0.05.

We firstly performed RT-PCR to determine whether LOC554202 was dysregulated in HCC. As shown in Fig. 1A, we found that HCC specimens exhibited a distinctly higher LOC554202 expression relative to the matched non-tumor tissues (*p* < 0.01). Furthermore, patients in stage (III–IV) presented a significantly higher LOC554202 expression relative to those in stages (I–II) (*p* < 0.01, Fig. 1B). Then, we performed ROC assays which showed that high LOC554202 expression had an AUC value of 0.7958 (95% CI: 0.7417–0.8499) for distinguishing HCC specimens from normal tissues (Fig. 1C) and had an AUC value of 0.7033(95% CI: 0.6180–0.7887) or distinguishing specimens with stages (III–IV) from specimens with stages (I–II) (Fig. 1D). Moreover, we observed that relative to the LO2 cells, the expressions of LOC554202 were distinctly higher in HCC cell lines like HepG2, Hep3B, Huh7, SMMC-7721, and LM3 (Fig. 1E, *p* < 0.01). Moreover, Adriamycin (ADM)-resistant LM3 and Huh7 cells displayed a distinctly higher expression of LOC554202 than corresponding parental cells (Fig. 1F, G). Overall, the abnormal expression of LOC554202 may affect the HCC progression and ADM resistance of HCC.

### Prognostic values of LOC554202 expression in HCC patients

For a better understanding of the clinical relevance of LOC554202 expressions in HCC, the 137 HCC patients were divided into two groups, group with a high expression (*n* = 70) and a group with a low expression (*n* = 67), according to the median expressions of LOC554202 in HCC samples. As presented in Table 2, high LOC554202 expression was associated with histologic grade (*p* = 0.025) and TNM stage (*p* = 0.008). Nevertheless, LOC554202 expression was not significantly related to other clinical features (All *p* > 0.05). Then, we collected 5-year survival data of 137 HCC patients and performed Kaplan–Meier analysis, finding that patients whose LOC554202 expression was high had shorter overall survival (OS, *p* < 0.0078; Fig. 1H) and disease-free survival (DFS, *p* < 0.0045; Fig. 1I) as compared with the LOC554202-low group. Univariate analyses revealed that TNM stage, histologic grade, as well as high LOC554202 expression could affect HCC patients’ survival (Tables 3 and 4). More importantly, Multivariate analysis confirmed that high LOC554202 expression could be used to independently predict HCC patients regarding the OS (HR = 2.873, 95% CI: 1.237–4.327, *p* = 0.011) and DFS (HR = 2.893, 95% CI: 1.231–4.443, *p* = 0.009). Overall, LOC554202 could be used to predict the prognosis of HCC patients.

**Table 2:** Correlations between LOC554202 and clinicopathologic variables of HCC.

Parameters	Group	Total	LOC554202 expression		*p*-value
			High	Low	
Gender	Male	77	37	40	0.420
	Female	60	33	27	
Age (years)	<50	71	34	37	0.661
	≥50	66	36	30	
Tumor size	<5	87	41	46	0.220
	≥5	50	29	21	
Tumor number	Solitary	73	34	39	0.258
	Multiple	64	36	28	
AFP	<200	64	30	34	0.355
	>200	73	40	33	
Hepatitis B	Negative	66	31	35	0.352
	Positive	71	39	32	
Histologic grade	High	54	34	20	0.025
	Low	83	36	47	
TNM stage	I–II	87	37	50	0.008
	III–IV	50	33	17

**Table 3:** Univariate and multivariate analysis of overall survival in HCC patients.

Variables	Univariate analysis			Multivariate analysis		
	HR	95% CI	*p-*value	HR	95% CI	*p*-value
Gender	0.782	0.323–1.784	0.328	–	–	–
Age	1.237	0.573–2.173	0.218	–	–	–
Tumor size	1.345	0.673–1.986	0.437	–	–	–
Tumor number	1.547	0.732–2.138	0.718	–	–	–
AFP	0.993	0.537–1.873	0.138	–	–	–
Hepatitis B	1.482	0.637–2.123	0.129			
Histologic grade	2.896	1.327–4.562	0.013	2.662	1.218–4.239	0.021
TNM stage	3.128	1.427–4.873	0.002	2.891	1.237–4.453	0.008
LOC554202 expression	3.051	1.327–4.642	0.008	2.873	1.237–4.327	0.011

**Table 4:** Univariate and multivariate analysis of disease-free survival in HCC patients.

Variables	Univariate analysis			Multivariate analysis		
	HR	95% CI	*p-*value	HR	95% CI	*p*-value
Gender	1.327	0.527–2.176	0.217	–	–	–
Age	1.423	0.632–1.893	0.137	–	–	–
Tumor size	1.443	0.549–2.016	0.213	–	–	–
Tumor number	1.632	0.732–2.218	0.328	–	–	–
AFP	1.231	0.652–1.893	0.213	–	–	–
Hepatitis B	0.982	0.632–2.328	0.138	–	–	–
Histologic grade	3.018	1.427–4.671	0.009	2.783	1.232–4.349	0.016
TNM stage	3.321	1.532–5.216	0.001	2.980	1.332–4.838	0.008
LOC554202 expression	3.113	1.438–4.673	0.004	2.893	1.231–4.443	0.009

### LOC554202 is activated by FOXO3 in HCC cells

**Fig. 2:**
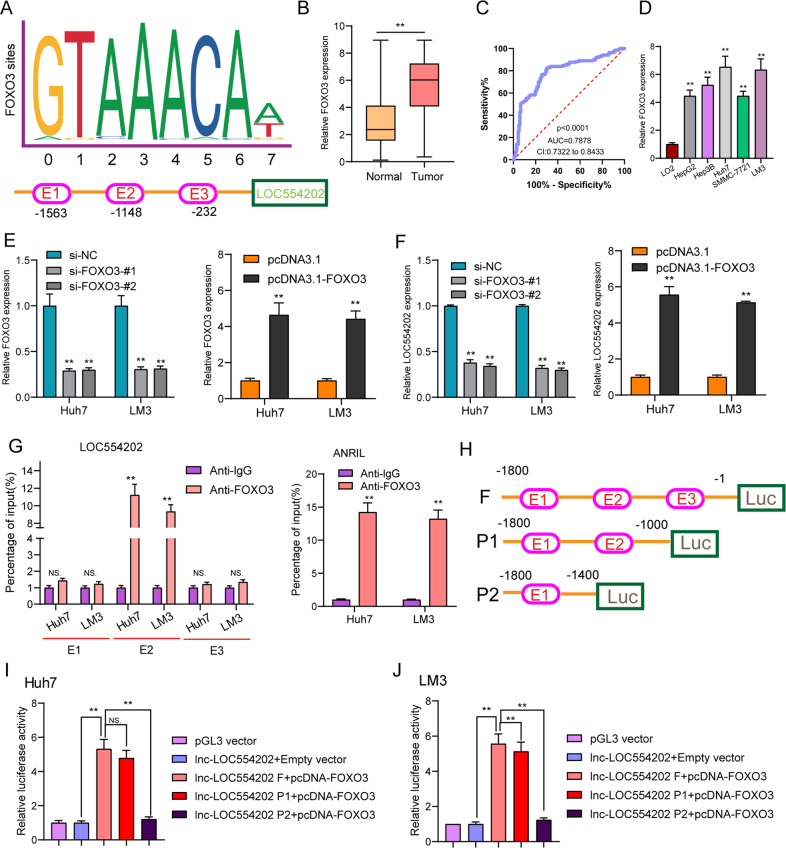
LOC554202 is activated by FOXO3 in HCC. **A** FOXO3-binding site prediction in the LOC554202 promoter region using JASPAR. **B** RT-PCR for the levels of FOXO3 in our cohort. **C** ROC curves of mRNA FOXO3 expression for differentiating advanced HCC tissue from early HCC tissue. **D** Relative expression of FOXO3 in five HCC cell lines and LO2. **E** The expression of FOXO3 in Huh and LM3 cells transfected with si-FOXO3 or pcDNA3.1-FOXO3. **F** RT-qPCR analysis of LOC554202 expression in Huh and LM3 cells after FOXO3 downregulation or upregulation. **G** ChIP-qPCR analysis of FOXO3 occupancy in the LOC554202 promoter in Huh and LM3 cells. **H** Construction of the luciferase reporter vector pGL3F, pGL3P1, and pGL3P2. **I**, **J** Relative luciferase activities as analyzed in Huh and LM3 cells after various transfection. *p < 0.05.

Although the distinct dysregulation of lncRNAs has been reported in several tumors, the regulators involved in the dysregulation of these molecules are not properly understood. In recent years, more and more studies indicated that several key transcription factors were involved in the overexpression of lncRNAs in tumor cells [8, 26]. Using JASPAR, which is an online tool for the exploration of transcription factors, we found several FOXO3-binding sites in the LOC554202 promoter regions with high scores (Fig. 2A). Then, we performed RT-PCR and found that FOXO3 presented an obviously upregulated level in HCC specimens from our cohort (Fig. 2B). ROC assays revealed that FOXO3 could be used to distinguish HCC tissues from non-tumor tissues with an AUC of 0.7878 (*p* < 0.001, Fig. 2C). FOXO3 was also highly expressed in five HCC cell lines (Fig. 2D). To explore whether the dysregulation of FOXO3 could influence LOC554202 expression, we performed RT-PCR using LM3 and Huh7 cells, which received the transfection of si-FOXO3-#1, si-FOXO3-#1, or pcDNA3.1-FOXO3, finding that knockdown of FOXO3 suppressed LOC554202 expression in the above cell lines, while overexpression of FOXO3 displayed an opposite trend (Fig. 2E, F). Subsequent ChIP assays documented an apparent FOXO3-binding activity on the endogenous LOC554202 promoter region around E2 (Fig. 2G). More importantly, based on luciferase reporter assays, FOXO3 only bound to the E2 (−1148 bp) binding site, and did not bind to the other two sites (Fig. 2H–J). Overall, our findings indicated that some TFs can contribute to the tumorigenesis and progression of various tumors not only via modulating the expression of protein-coding genes but also through affecting lncRNAs transcription.

### LOC554202 loss of function markedly impairs HCC cell malignant phenotypes

**Fig. 3:**
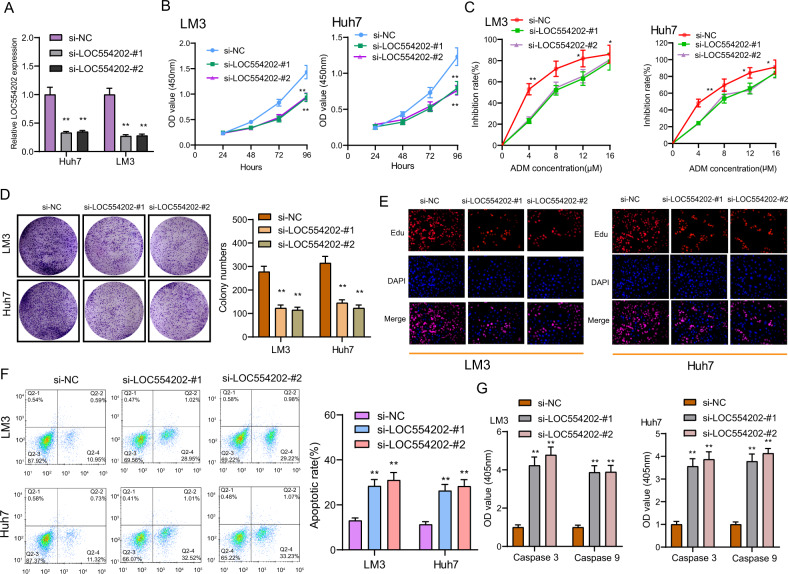
The effect of LOC554202 on HCC cell proliferation and apoptosis. **A** Oligonucleotides targeting LOC554202 (si-LOC554202-#1 and si-LOC554202-#2) and negative controls (si-NC) were transfected into Huh and LM3 cells. **B** CCK-8 assays of Huh and LM3 cells after transfection. **C** Huh and LM3 cells were treated with ADM (0, 4, 8, 12, 16 μM) for 48 h. The inhibition rate increased with the increase of concentration of ADM. **D** Knockdown of LOC554202 suppressed colony-formation as demonstrated by colony-formation assays in HCC cells. **E** Cell proliferation was evaluated using EdU incorporation assays. **F** Flow cytometry assays were applied to detect cell apoptosis in si-LOC554202-#1 and si-LOC554202-#2 transfected Huh and LM3 cells. **G** The activities of caspase 3/9 were determined. *p < 0.05, **p < 0.01.

**Fig. 4:**
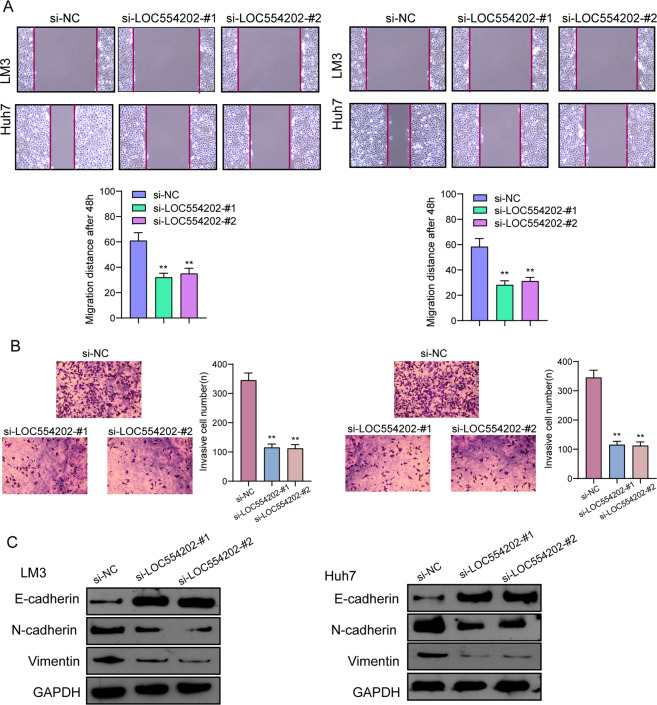
Knockdown of LOC554202 suppressed the migration and invasion of HCC cells. **A** Wound-healing assay was used to detect cell migration in si-LOC554202-#1 and si-LOC554202-#2 transfected Huh and LM3 cells. **B** The invasion abilities of tumor cells were assessed using transwell assays. **C** The influences of LOC554202 knockdown on EMT-related factors in Huh and LM3 cells were respectively analyzed using western blot. *p < 0.05, **p < 0.01.

To determine the function possessed by LOC554202 in HCC cells, LM3 and Huh7 cells were used to construct stable cells with LOC554202 knockdown due to the higher expression of LOC554202 in the above two cells (Fig. 3A). Next, as shown in CCK-8 assays, LOC554202 knockdown suppressed the proliferation of LM3 and Huh7 cells (Fig. 3B). We also explored the effect of LOC554202 on chemotherapy resistance of HCC cells, finding that inhibition rate increased with the increase of concentration of ADM, and knockdown of LOC554202 decreased the inhibition effect of ADM on HCC cells (Fig. 3C). Also, based on the clone formation assays, LM3 and Huh7 cells presented a decreasing clone forming ability due to LOC554202 knockdown (Fig. 3D). Besides, as confirmed by the 5-ethynyl-2′-deoxyuridine (EdU) proliferation assay, LOC554202 depletion caused the cell to grow slowly (Fig. 3E). Importantly, we also showed that knockdown of LOC554202 promoted the apoptosis of LM3 and Huh7 cells (Fig. 3F). On the other hand, we also demonstrated that the activity of Caspase 3/9 was suppressed in HCC cells transfected with si-LOC554202-#1 and si-LOC554202-#2 (Fig. 3G). On the other hand, we explored the possible function of knockdown of LOC554202 in the metastatic potential of HCC cells. As presented in Fig. 4A, using the wound-healing assays, we observed that LOC554202 depletion caused significant inhibition of HCC cell migration. Besides, according to the transwell assays results, LOC554202 knockdown suppressed the invasive ability of LM3 and Huh7 cells compared with control cells (Fig. 4B). Since epithelial–mesenchymal transition (EMT) progress could affect the migration and invasion ability, the possible functions of LOC554202 on the EMT process were studied. As expected, LOC554202 knockdown inhibited the EMT process in HCC cells characterized by decreased expressions of mesenchymal marker (Vimentin and N-cadherin) and increased expressions of epithelial marker (E-cadherin) (Fig. 4C). Overall, our findings suggested LOC554202 as an oncogenic lncRNA in HCC progression.

### LOC554202 directly interacts with miR-485-5p in HCC cells

**Fig. 5:**
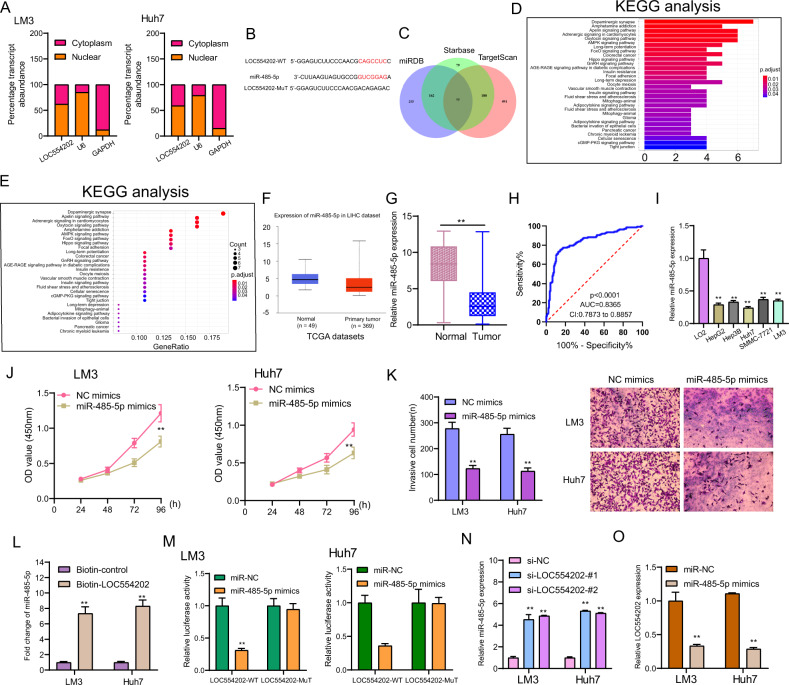
LOC554202 competitively sponged miR-485-5p. **A** Relative LOC554202 expression levels in nuclear and cytosolic fractions of Huh and LM3 cells. **B** The schematic diagram presents the complementary binding sites within LOC554202 and miR-485-5p. **C** Venn analysis of the potential targeting genes of miR-485-5p in miRDB, Starbase, and TargetScan. **D**, **E** KEGG pathways for the above potential targeting genes. **F** The dysregulated expression of miR-485-5p in HCC tissues in TCGA data sets. **G** The distinct upregulation of miR-485-5p expression in HCC specimens from our cohort. **H** ROC assays for the diagnostic value of miR-485-5p expression in distinguishing HCC specimens. **I** The expression of miR-485-5p in HCC cell lines and LO2. **J** After overexpression of miR-485-5p, HCC cell proliferation was determined by CKK-8 assays. **K** After overexpression of miR-485-5p, the invasion ability of HCC cells was determined by transwell assays. **L** RNA-pulldown for the exploration of the association between LOC554202 and miR-485-5p. **M** Overexpression of miR-485-5p decreased the Luciferase activity of wild-type MSI1 3′ UTR construct in both Huh and LM3 cells. **N** Knockdown of LOC554202 suppressed the expression of miR-485-5p. **O** Overexpression of miR-485-5p inhibited the levels of LOC554202, which was determined by RT-PCR. *p < 0.05, **p < 0.01.

As confirmed by studies, lncRNAs function as the endogenous RNAs competing for microRNAs, thereby regulating the underlying targets [27]. Subcellular fractionation assays assisted in confirming the potential mechanisms regarding the LOC554202-related modulation of biological features in HCC cells. As shown in Fig. 5A, we observed that LOC554202 was distributed in both the cytoplasm and nucleus. Using starbase, we predicted that LOC554202 could be targeted by miR-485-5p (Fig. 5B). Moreover, Kyoto Encyclopedia of Genes and Genomes (KEGG) assays revealed that the potential targeting genes of miR-485-5p (*n* = 77) were positively associated with tumor-related pathways, suggesting it as a functional regulator in the progression of HCC (Fig. 5C–E). Then, we determined miR-485-5p expression in HCC in TCGA data sets, finding miR-485-5p expression exhibited a decreased trend in HCC specimens relative to non-tumor liver tissues (Fig. 5F). In our cohort, we further confirmed that miR-485-5p expression in HCC specimens was distinctly lower than that in matched non-tumor specimens(Fig. 5G). The diagnostic assays using the expression data from our cohort revealed that high miR-485-5p expression had an AUC value of 0.8365 (95% CI: 0.7873–0.8857) for distinguishing HCC specimens from normal tissues (Fig. 1H). In addition, the distinct downregulation of miR-485-5p also occurred in Five HCC cell lines (Fig. 5I). As found by the functional assays, miR-485-5p overexpression significantly hindered LM3 and Huh7 cells in terms of proliferation and invasion according to the results of CCK-8 assays and transwell assays (Fig. 5J, K). For figuring out how LOC554202 is associated with miR-485-5p, RNA-pulldown assays were conducted, finding that LOC554202 had the function of precipitating miR-485-5p in HCC cells (Fig. 5L). The luciferase assay showed that miR-485-5p mimic could exert an obvious inhibiting effect on the reporter activities of wt-LOC554202 but not mut-LOC554202 in LM3 and Huh7 cells (Fig. 5M). Moreover, we further performed RT-PCR to confirm the above results and found that knockdown of LOC554202 promoted the expression of miR-485-5p (Fig. 5N), while miR-485-5p overexpression led to a decreased expression of LOC554202 in LM3 and Huh7 cells (Fig. 5O). Overall, our findings strongly suggest that LOC554202 targeted and negatively regulated miR-485-5p in HCC cells.

### LOC554202 modulated malignant behaviors of HCC via miR-485-5p/BSG axis

**Fig. 6:**
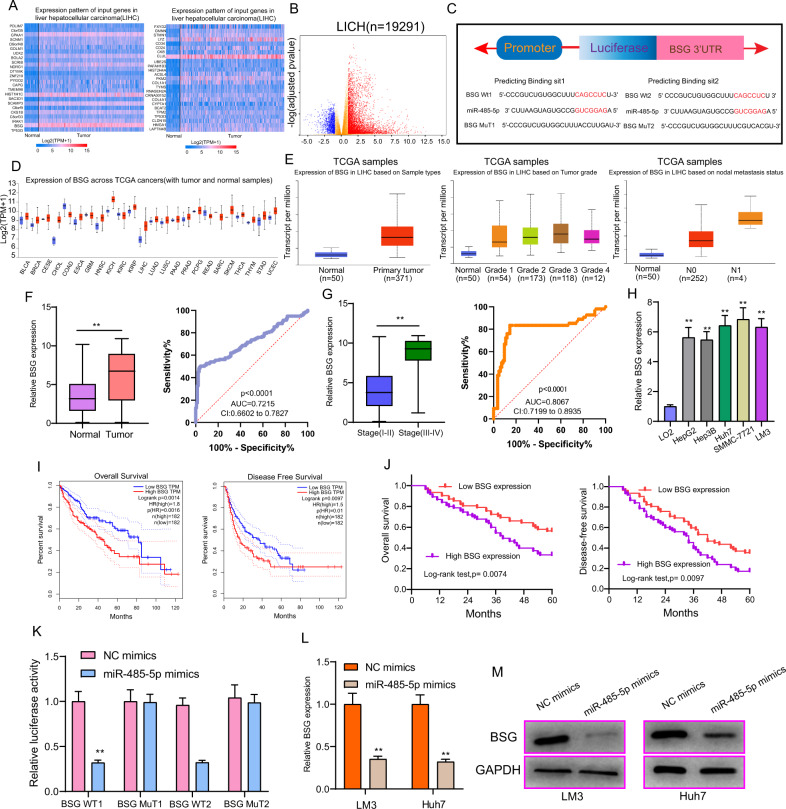
BSG was a target gene for miR-485-5p. **A** The top 50 upregulated lncRNAs in HCC tissues were shown in a heat map using TCGA data sets. **B** Volcano plots show differentially expressed lncRNAs in HCC tissues by analyzing TCGA data sets. **C** The predicted binding sites of miR-485-5p within BSG were shown. **D** Pan-cancer analysis of BSG expression using TGCA data sets. **E** The expression pattern of BSG in HCC tissues with different clinical grades and with or without metastasis by analyzing TGCA data sets. **F** The distinct upregulation of mRNA BSG expression in HCC and its diagnostic significance in our cohort. **G** Higher levels of mRNA BSG in HCC specimens and its diagnostic value were observed. **H** Relative expression of mRNA BSG in five HCC cell lines and LO2. **I** The prognostic value of high BSG expression was determined in HCC patients, which was analyzed using GEPIA. **J** Survival analysis of 137 HCC patients by Kaplan–Meier methods. **K** Relative luciferase activity was analyzed in Huh and LM3 cells. **L**, **M** Overexpression of miR-485-5p suppressed the expression of BSG at mRNA and protein levels. *p < 0.05, **p < 0.01.

**Fig. 7:**
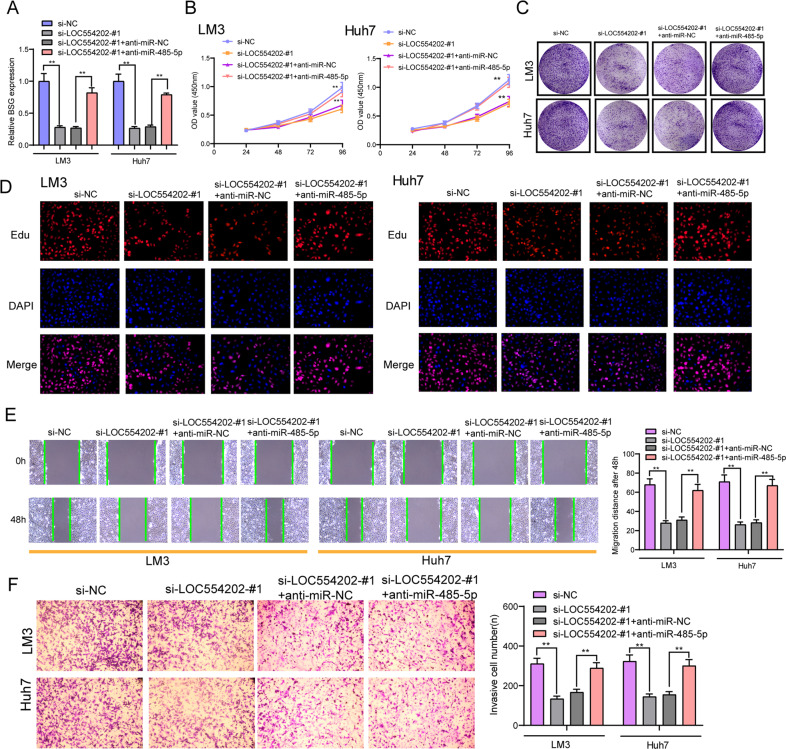
Deficiency of miR-485-5p attenuates the regulatory effect of LOC554202 knockdown on the progression of HCC cells. **A** The expression levels of BSC in LM3 and Huh7 cells after knockdown of LOC554202 and/or inhibition of miR-485-5p. The CCK-8 assays (**B**), colony-formation assays (**C**), Edu assays (**D**), Cell invasion (**E**), and migration (**F**) assays following knockdown of LOC554202 and/or inhibition of miR-485-5p. *p < 0.05, **p < 0.01.

**Fig. 8:**
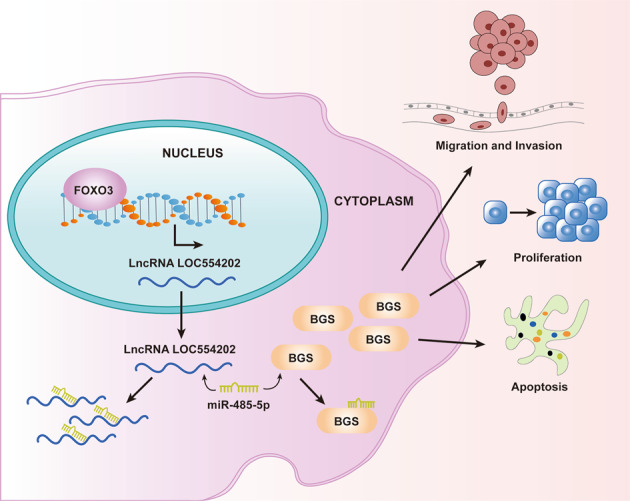
Hypothetical model for LOC554202 function in HCC. LOC554202 served as a sponge of miR-485-5p to increase BSG expression, thus further promoting the progression of HCC.

We first predicted the potential targets regarding miR-485-5p, for further investigating the function exhibited by miR-485-5p in HCC. Using TCGA data sets with UALCAN, we identified the top 50 overexpressed lncRNAs in HCC, which was shown in the heat map (Fig. 6A). In addition, the dysregulated genes analyzing TCGA data sets were shown in the Volcano plot (Fig. 6B). Then, we searched mi-Randa, Starbase, and TargetScan, finding that miR-485-5p may directly target BSG which was among the top 50 overexpressed lncRNAs in HCC (Fig. 6C). Previous studies reported the functional role of BSG in the development of many tumors [28, 29]. However, the potential functions of BSG in HCC remained largely unclear. Using UALCAN, we performed pan-cancer expression, finding that BSG exhibited a high level in many types of tumors based on the TCGA data sets (Fig. 6D). Then, we also observed the high expression of BSG and its positive relation to advanced stages and tumor metastasis (Fig. 6E). In our cohort, we also confirmed the mRNA expression of BSG was upregulated in HCC specimens and had a potential diagnostic in distinguishing HCC specimens from non-tumor specimens (Fig. 6F). Besides, we performed a similar analysis, finding that BSG exhibited a higher level in HCC specimens than in non-tumor tissues and its diagnostic value was also confirmed (Fig. 6G). Moreover, we provided evidence that BSG mRNA exhibited an obviously upregulated expression in five HCC cell lines (Fig. 6H). On the other hand, we explored the prognostic value of BSG expression in HCC patients. 137 HCC patients were divided into two groups, a group with a high expression (*n* = 76) and a group with a low expression (*n* = 61), according to the median expressions of BSG in HCC samples. Based on the results of GEPIA, we observed that patients whose BSG expression was high exhibited a shorter OS (*p* = 0.0014) and DFS (*p* = 0.0097) relative to patients whose BSG expression was low (Fig. 6I), which was also confirmed in our cohort (Fig. 6J). As suggested by those finding, together with previous findings, BSG could be a tumor promoter in HCC. Importantly, based on luciferase reporter assays, miR-485-5p overexpression remarkably suppressed the BSG wild-type (wt) luciferase reporters (Fig. 6K). Furthermore, miR-485-5p overexpression in LM3 and Huh7 cells significantly lowered the mRNA and protein levels of BSG (Fig. 6L, M). On that account, miR-485-5p may suppress the progression of HCC via targeting BSG. To further explore whether miR-485-5p/BSG axis could affect the HCC progression mediated by LOC554202, si-NC, si-LOC554202-#1, si-LOC554202-#1, and anti-miR-NC or anti-miR-485-5p were used to transfect the LM3 and Huh7 cells. As presented in Fig. 7A, BSG levels decreased due to LOC554202 knockdown and it was obviously reduced by transfection of anti-miR-485-5p. Besides, the proliferation, migration, and invasion suppressions caused by silencing LOC554202 were distinctly attenuated by miR-485-5p exhaustion in LM3 and Huh7 cells (Fig. 7B–F). Taken together, our study revealed that FOXO3-induced upregulation of LOC554202 exerted its function as a ceRNA through sponging miR-485-5p to regulate downstream BSG expression, and therefore contributed to the proliferation and metastasis of HCC cells (Fig. 8).

## Discussion

In recent years, an increasing number of lncRNAs have emerged as critical regulators in tumor progression [30, 31]. However, the function of many lncRNAs remains unclear in HCC. In this study, we identified a possible HCC-related lncRNA, LOC554202 which was confirmed to exhibit an obvious upregulation in HCC tissues and HCC/ADM cells, indicating that LOC554202 may contribute to chemotherapy resistance. The diagnostic value of LOC554202 in distinguishing HCC specimens from non-tumor specimens was also confirmed. Clinical assays revealed that high LOC554202 expression was associated with advanced clinical progress and poor prognosis of HCC patients. In addition, we performed functional assays, finding that LOC554202 knockdown hindered HCC cells regarding the proliferation, migration, and invasion while facilitated the apoptosis. Therefore, the findings in the study proved that LOC554202 may be used as a novel biomarker to predict the diagnosis and prognosis of HCC patients, and also a therapeutic target.

Although studies have confirmed the abnormal expressions of lncRNAs in different tumors, the potential mechanisms involving lncRNAs dysregulation in tumor cells remained unclear. Interestingly, many studies have proved that some TFs, as well as epigenetic regulators, could affect the transcription of some lncRNAs [32]. For instance, STAT1-mediated lncRNA LINC00174 overexpression promoted the proliferation and invasion regarding colorectal carcinoma cells via regulating the miRNA-1910-3p/TAZ axis [33]. HIF1-induced upregulation of lncRNA RAET1K sponged miRNA-100-5p, thus promoting HCC cells in terms of their proliferation and metastasis [34]. In this study, FOXO3 was predicted to be bound to the LOC554202 promoter region by the use of the JASPAR online database. Previously, FOXO3 overexpression and its oncogenic roles were reported in many tumors, including HCC [15, 35]. Mechanism experiments demonstrated that FOXO3 could bind to the LOC554202 promoter region and induce its transcription. Thus, our findings revealed FOXO3 may promote HCC progression via upregulating LOC554202.

In recent years, the expression and function of LOC554202 have been reported in several tumors. For instance, high LOC554202 expression was reported to partially represent the poor prognosis of colorectal cancer patients as well as could predict a favorable outcome in patients treated with oxaliplatin [36]. LOC554202 was shown to be highly expressed in lung cancer and promote acquired gefitinib resistance in lung cancer via sponging miRNA-31 expressions [21]. In laryngeal squamous cell carcinoma, based on studies, high LOC554202 levels could regulate miRNA-31, thereby promoting tumor cells in terms of proliferation and metastasis [22]. However, whether LOC554202 may influence HCC progression had not been reported. Given the distinct overexpression of LOC554202 in HCC and its clinical value, we further performed loss-of-function assays to explore the potential effects of LOC554202 on HCC cells, finding that knockdown of LOC554202 suppressed the proliferation, metastasis, and EMT progress of HCC cells. Overall, our findings suggested LOC554202 as an oncogenic lncRNA in HCC progression.

It has been confirmed that one of the major functions of lncRNAs is to harbor miRNAs to act as a “sponge” and decrease the expressions and activities of miRNAs [37, 38]. For figuring out the potential molecular mechanism for LOC554202 to regulate the downstream functional factors in HCC, the localization of LOC554202 in LM3 and Huh7 cells was first identified because the potential functions of lncRNAs were associated with their subcellular localization. The results of subcellular fractionation revealed that LOC554202 was expressed in the cytoplasm and nucleus. StarBase v2.0 indicated that LOC554202 may be a target of miR-485-5p. In previous studies, miR-485-5p was reported to promote the progression of several tumors, including HCC, which was also demonstrated in our functional assays when miR-485-5p was overexpressed [39, 40]. In addition, in HCC, several lncRNAs such as lncRNA RHPN1-AS and LINC00460 were reported to promote the proliferation and invasion of HCC cells via sponging miR-485-5p [39, 41]. These findings suggested miR-485-5p as a common target of different lncRNAs. In this research, we also provided evidence that LOC554202 acted as a ceRNA for miR-485-5p in the cytoplasm and sponged miR-485-5p. Thus, our findings suggested that LOC554202 may promote HCC progression via sponging miR-485-5p.

Growing basic evidences have indicated that basigin (BSG, also named TCSF, CD147, EMMPRIN) is a transmembrane-type glycoprotein that is strongly expressed on the cell membranes of several tumor cells [42]. It has been confirmed that BSG could stimulate para-cancerous fibroblast secreting matrix metalloproteinases (MMP) which can help cancer cells easily penetrate the basement membrane of blood vessels, therapy resulting in metastasis [43]. Previously, large-scale studies have found the obvious BSG upregulation in many tumors [44, 45]. Importantly, BSG has been reported to promote the proliferation and invasion of HCC cells [46]. In this study, we also observed that BSG was highly expressed in HCC and predicted a poor prognosis of HCC patients. Interestingly, by the use of bioinformatics analysis, we found that BSG may act as a targeting protein of miR-485-5p, which was confirmed through the luciferase assays. Moreover, BSG was overexpressed by LOC554202, while downregulated by miR-485-5p. In addition, rescue experiments further confirmed knockdown of miR-485-5p reversed the oncogenic effects of LOC554202 overexpression on the proliferation, migration, and invasion of HCC cells. Overall, our findings revealed that FOXO3-induced upregulation of LOC554202 exhibited its oncogenic effects at least in part by modulating the miR-485-5p/BSG axis. The FOXO3/LOC554202/miR-485-5p/BSG axis may be a promising therapeutic target for HCC.

## Conclusions

The study is the first one that showed the distinct upregulation of LOC554202 expression in HCC specimens, which is related to the poor clinical outcome and can be used to predict the diagnosis and prognosis of HCC patients. FOXO3 can be directly bound to the LOC554202 promoter region, thereby activating its transcription. Silencing LOC554202 can target the miR-485-5p/BSG axis, thus hindering the proliferation and metastasis of HCC cells and inducing apoptosis. To sum up, the data in the study prove the possibility of targeting LOC554202/miR-485-5p/BSG axis being an effective alternative treatment, which can provide an essential implication for HCC treatment and diagnosis.

## Data Availability

The data used to support the findings of this study are available from the corresponding author upon request
